# The control strategy for distributed energy storage devices using fully diffusion strategy

**DOI:** 10.1016/j.heliyon.2025.e42175

**Published:** 2025-01-23

**Authors:** Zejian Liu, Ping Yang, Xu Lin, Ziwei Fan, Cencen Hong

**Affiliations:** a Key Laboratory of Clean Energy Technology of Guangdong Province, School of Electric Power Engineering, South China University of Technology, Guangzhou, 510640, China; b Power Dispatch Control Center, Guangdong Power Grid Corporation, Guangzhou, 510699, China; c Shenzhen Huagong Energy Technology Co., Ltd, Shenzhen, 518100,China; d School of Automation, Nanjing Institute of Technology, Nanjing, 211167, China

**Keywords:** Diffusion strategy, Energy storage device unit (ESU), State-of-charge (SOC) equalization, Voltage compensation

## Abstract

The distributed energy storage device units (ESUs) in a DC energy storage power station (ESS) suffer the problems of overcharged and undercharged with uncertain initial state of charge (SOC), which may reduce the service period of ESUs. To address this problem, a distributed secondary control based on diffusion strategy is proposed. In the first layer, each ESUs operates with its local controller by droop control. In the second layer controller, diffusion strategy coordinate the SOC of multiple distributed ESUs with uncertain initial SOC. Compared with centralized control, the proposed method contributes to a higher scalability and reliability. Diffusion strategy allows adjacent nodes to diffuse and cooperate information in real-time, and it includes a stochastic gradient term. Thus, diffusion strategy can achieve a higher convergence rate and lower mean-square-error than consensus strategies. For the second layer controller, first the coordinated control of ESUs is transformed into two optimization problems: the balanced output voltage and the balanced SOC of each ESU. Then, diffusion strategy is used to solve the optimization problems. Finally, a simulation model is built to verify the effectiveness of diffusion strategy. The results show that diffusion strategy always ensure the stability irrespective of the combination topology. Compared with the widely used consensus method, diffusion strategy shows a good performance in realizing demand response for distributed ESUs.

## 1 Introduction

Renewable energy generation systems, such as offshore wind power generation system and photovoltaic power generation system are effective measures to solve the problems of ever-deteriorating environment and impending depletion of fossil fuels [[1], [2], [3], [4]]. For the features of renewable energy, the generated output power is random and intermittent. Thus, to increase the accommodation and the utilization of wind energy, an energy storage power station (ESS) is configured to realize peak shaving for the bulk power grid system [5,6].

Compared with alternating current ESS, direct current ESS offers the advantages of high quality power energy with lower harmonic current, no transformer loss, high transmission capacity, and so on. Thus, an offshore wind farm should be configured with a DC ESS to balance the local power and output power [7,8].

In the DC ESS, the distributed ESUs can apply the centralized control method and decentralized control method [9]. Using the centralized control method, the superior centralized controller is necessary to process massive data, and the data transmission relies on the performance of communication system. The fault of communication system and centralized controller may lead to the entire ESS down, which results in a poor fault tolerant ability [10].

Using the decentralized control method, each ESU is configured with its unique controller, the single individual controller's fault has no influence on the other ESUs. And it only detects the information of local ESU with no need for communication system. Thus, the decentralized control system is more reliable [11,12]. While, in general, the decentralized control system adopts droop control, which results in a steady-state errors of the output voltage [13].

The distributed control method overcomes the shortcomings of both decentralized control system and centralized control system [14]. And the distributed control method only requires the local information and the adjacent nodes' information to implement that the state variables of each ESSs tend to be consistent. Thus, the distributed control method is more scalability, flexibility, and robustness [15]. Among the distributed multi-agent algorithms, the consensus algorithm is widely used, but its convergence rate is small, and the convergence time is long. While the diffusion strategy adopts gradient algorithm to improve convergence rate and reduce the convergence time [16,17].

Micro grid and smart grid have widely used the distributed consensus algorithm in many fields, such as the load power allocation, economic dispatch control, the frequency and voltage control and so on [[18], [19], [20], [21], [22]]. For instance Ref. [20], applies the dominated group search optimization algorithm to achieve the targets in an economic environmental manner of cryogenic energy storage system in microgrids.

For ESS [[23], [24], [25], [26]], introduce a two-level control framework, the first layer controller applies a droop control, the second layer controller applies the distributed consensus algorithm to compensate voltage deviation correction caused by the first layer controller. But the consensus algorithm contains an integration element, the initial value of integration may cause a longer convergence time and steady-state error.

The first layer controller with droop control ensures that ESS can operates with individual controllers. But the traditional droop control strategy always adopts constant droop coefficient, which leads to the unbalanced SOC [19]. Once the initial SOCs of each ESUs are different, and the line impedances are also different, a portion of ESUs may come into being serious problems, such as over-discharging and over -charging. And affected by the line impedances, the output voltages of each ESUs are not the same, which results in a circulating current. With a larger droop coefficient, the influence of line impedances can be neglected, while the steady-state error by droop control is relatively large. And The second layer controller with distributed consensus algorithm can address the problems caused by the different initial SOCs and line impedance.

Thus, in this paper, a two layer control framework is introduced, and the first layer controller adopts an improved droop control with adaptive droop coefficients, which can adjust the SOCs of each ESUs balanced during charging process and discharging process. The second layer controller adopts the distributed diffusion strategy to compensate the voltage error caused by the line impedances and the first layer controller.

Compensating the voltage error is equivalent to an optimization problem. One of the optimization objectives is to minimize the variance of the average output voltages of each ESUs, it is equivalent to compensate the voltage error caused by line impedance. The other one optimization objective is to minimize the variance of the output power of each ESUs, it is equivalent to compensate voltage error caused by droop control. In other words, using the distributed consensus algorithm, one control target is balanced power allocation among each ESUs to achieve equalization SOC, and the other control target is voltage deviation correction caused by droop control to achieve a rated reference DC bus voltage.

Finally, a simulation model is built to verify the effectiveness of proposed two layer control framework.

## 2 The topology of distributed ESU

### 2.1 The configuration of ESS

**Fig. 1:**
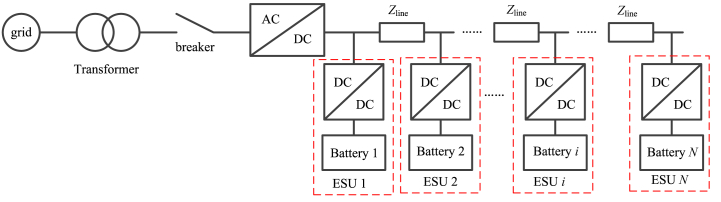
The configuration of ESS.

The ESS for offshore wind farm consists of transformer, circuit breaker, bi-direction DC/DC converter, bi-direction AC/DC converter, and battery. Fig. 1 shows the configuration of ESS.

**Fig. 2:**
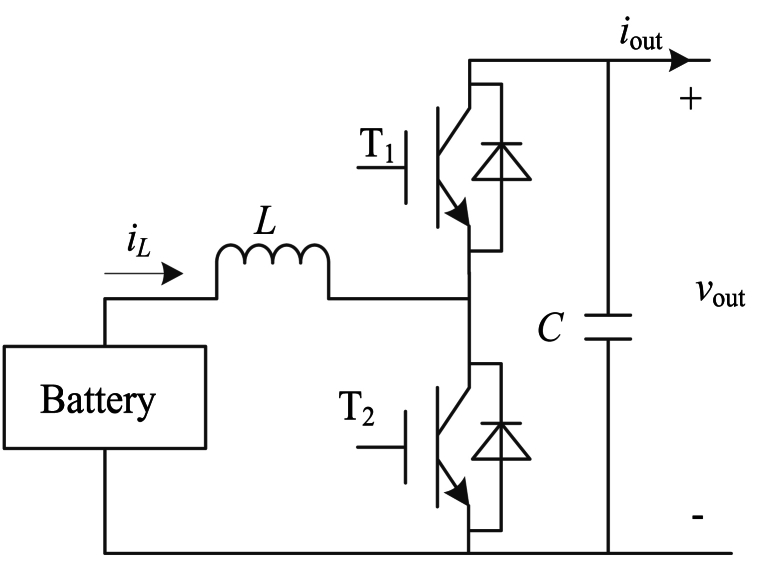
The ESU

In Fig. 1, *Z*_line_ is the line impedance. The battery can use different types of battery with different capacity, such as lithium Battery, super capacitor, flywheel energy storage system and so on. ALL the battery can be with different discharging and charging power. DC/DC converter applies the Buck/Boost converter. An ESU contains a battery and DC/DC converter, and it is plug-and-play. The ESU is shown in Fig. 2.

In Fig. 2, *v*_out_, *i*_out_ are the output voltage and output current of ESU, *L* is the inductance, T_1_ and T_2_ are insulated-gate bipolar transistors (IGBT), *C* is the capacitance. *i*_*L*_ is the output current of battery.

### 2.2 The control strategy for ESU

**Fig. 3:**
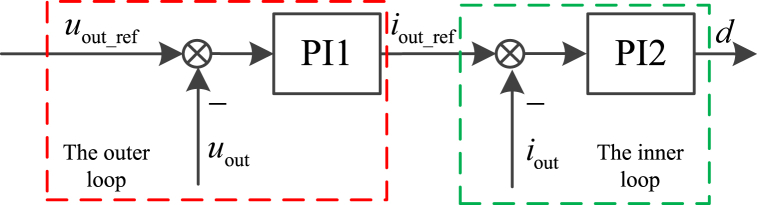
The control block diagram.

For any individual ESU, the control strategy contains two control loops, and it is divided into some categories, such as constant voltage control strategy, constant current control strategy, and so on. Using the constant voltage control strategy, the detailed control block diagrams is shown in Fig. 3.where, *u*_out_ref_, *i*_out_ref_ is the reference of output voltage and output current of ESU, *d* is the duty cycle for IGBT in Buck/Boost converter. Both PI1 and PI2 are proportional-integral controllers. And *i*_out_ref_ is expressed as Equation (1), *d* is expressed as Equation (2).

As shown in Fig. 3, it consists of two control loops, the input of outer loop controller is the difference value between the output voltage reference and the actual output voltage, and the output of outer loop controller is output current reference. And the input of inner loop controller is the difference value between the output current reference and actual output current, the output of inner loop controller is duty cycle. *I*_out_ref_ and *d* are expressed as

(1) $$i_{\text{out}\_\text{ref}}=k_{P1}\left(u_{\text{out}\_\text{ref}}-u_{\text{out}}\right)+k_{I1}\int \left(u_{\text{out}\_\text{ref}}-u_{\text{out}}\right)dt$$

(2) $$d=k_{P2}\left(i_{\text{out}\_\text{ref}}-i_{\text{out}}\right)+k_{I2}\int \left(i_{\text{out}\_\text{ref}}-i_{\text{out}}\right)dt$$

where, *k*_P1_ and *k*_I1_ are the proportional coefficient and integral coefficient for PI1 controller, *k*_P2_ and *k*_I2_ are the proportional coefficient and integral coefficient for PI2 controller.

## 3 The first layer controller

### 3.1 The principle of droop control

**Fig. 4:**
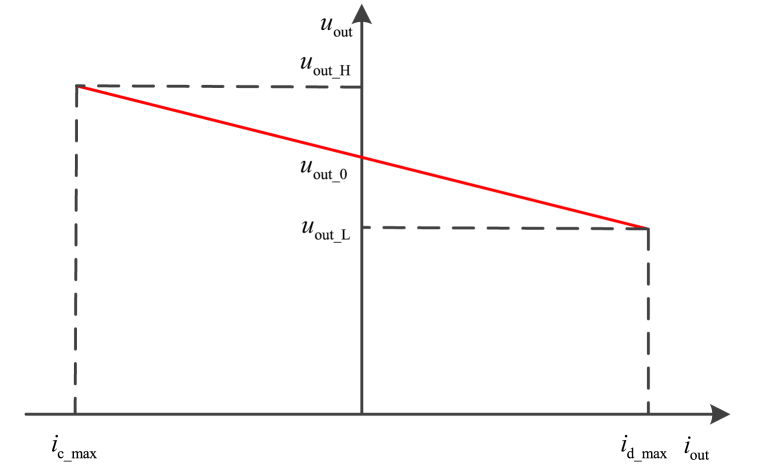
The droop coefficient curve for ESU.

The droop control for ESU is different from the droop control for photovoltaic power generation system and wind power generation system. The two-quadrant working principle of ESU is shown in Fig. 4. In the first quadrant, ESU operates in discharging mode, and ESU outputs the power to DC bus. In the second quadrant, ESU operates in charging mode, and DC bus outputs the power to ESU.

In Fig. 4, *u*_out_H_, *u*_out_L_ is the maximum output voltage and the minimum output voltage of ESU, *u*_out_0_ is the no-load output voltage, *i*_d_max_ and *i*_c_max_ are the maximum discharging current and charging current.

The function expression of droop control is described as Equation (3).

(3) $$u_{\text{out}\_\text{ref}}=u_{\text{out}\_0}-R\times i_{\text{out}}$$

where *R* is the droop coefficient. Thus, the output power of ESU is inversely proportional to its SOC.

The different initial SOCs of each ESUs may result in that partial ESUs may be overcharged and over discharged.

### 3.2 The improved droop control

ESUs in the ESS should support plug and play functionality. And the capacities, SOCs and rated powers of multiple distributed ESUs may be different. To adjust the SOCs of each distributed ESUs balanced, an improved droop control with adaptive droop coefficients is proposed.

For the charging process, the droop coefficient is expressed as

(4) $$R_{i\_c}=\frac{C_{\max}}{C_{i}}\times R_{i}\times \text{SO}{C_{i}}^{n}$$

For the discharging process, the droop coefficient is expressed as

(5) $$R_{i\_d}=\frac{C_{\max}}{C_{i}}\times R_{i}\times \text{SO}{C_{i}}^{-n}$$

where, *R*_*i*_ is the constant droop coefficient of the *i*th ESU, *R*_*i*_c_ is the adaptive droop coefficient of the *i*th ESU during the charging period, *R*_*i*_d_ is the adaptive droop coefficient of the *i*th ESU during the discharging period. *n* is the equalizing coefficient of SOC, and it is a positive constant. *C*_max_ is the maximum capacity of ESU among all the distributed ESUs, *C*_*i*_ is the capacity of the *i*th ESU. SOC_*i*_ is SOC of the *i*th ESU.

Based on Equation (4) and Equation (5), the adaptive droop coefficients are related to SOCs of each ESUs. And according to Equation (5), during the discharging period, since *n* is a positive value, SOC_*i*_^-*n*^ is a monotone decreasing function of SOC_*i*_. Hence, with the larger initial SOC, the droop coefficient is small, which contributes to a higher discharging current. Conversely, with the smaller initial SOC, the droop coefficient is larger, which contributes to a lower discharging current. Therefore, the SOC of ESU with larger initial SOC falls faster than the SOC of ESU with smaller initial SOC.

Based on Equation (4), during the charging period, SOC_*i*_^*n*^ is a monotonic increasing function of SOC_*i*_. Hence, with the larger initial SOC, the droop coefficient is large, which contributes to a lower charging current. Conversely, with the smaller initial SOC, the droop coefficient is small, which contributes to a higher charging current. Therefore, the SOC of ESU with larger initial SOC increases more slowly than the SOC of ESU with smaller initial SOC.

That is, with the adaptive droop coefficients, the SOCs of each distributed ESUs are adjusted to be balanced during the discharging and charging process. And the improved droop control of each ESUs only detect its own information without communication with other ESUs. Thus, the communication network system is unessential.

In addition, according to Equation (4) and Equation (5), the balanced speed of SOC is related to *n*. The larger *n* contributes to a larger balanced speed. While with a higher *n*, the initial discharging and charging power of ESU is larger. Thus, *n* is set according to two aspects: the balanced speed, the maximum discharging and charging power.

**Fig. 5:**
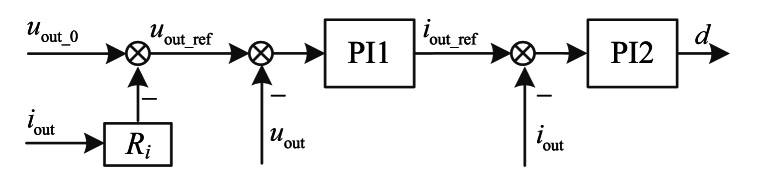
The improved droop control.

The control schematic diagram of proposed droop control is shown in Fig. 5.

### 3.3 The technical limitation of improved droop control

**Fig. 6:**
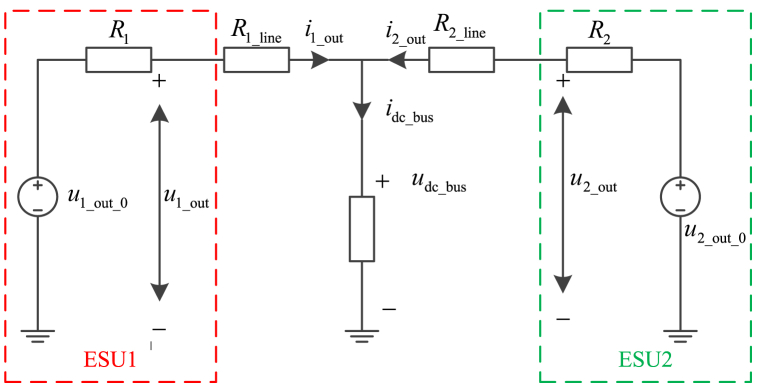
The equivalent circuit of two distributed ESUs.

Since the existence of line impedances, the DC bus voltages at each nodes are different, and it cannot be calculated by Equation (3). Fig. 6 shows the equivalent circuit of an example with two ESUs.

As shown in Fig. 6, the DC bus voltage is expressed as Equation (6).

(6) $$u_{\text{dc}\_\text{bus}}=u_{1\_\text{out}\_0}-\left(R_{1}+R_{1\_\text{line}}\right)\times i_{1\_\text{out}}=u_{2\_\text{out}\_0}-\left(R_{2}+R_{2\_\text{line}}\right)\times i_{2\_\text{out}}$$

where *u*_dc_bus_ is the voltage of DC bus, *R*_1_line_ and *R*_2_line_ are the line impedance.

According to Equation (6), for the existence of line impedances, there is a circulating current in two ESUs, and the output voltages of ESUs are different, too. Affected by the operation condition and ambient environment, the line impedance changes during the operation period.

With a larger droop coefficient, the influence of line impedance is neglected. While there is a steady-stable error by using droop control, and with a larger droop coefficient, the steady-stable error increases, which results in a DC bus voltage drop. Thus, the droop coefficient is limited by Equation (7).

(7) $$0<R_{i}<\frac{\Delta u_{\text{dc}\_\max}}{{i_{i\_\text{out}}}_{\_\max}}$$

where $\Delta u_{\text{dc}\_\max}$ is the maximum fluctuation of DC bus voltage, *i*_*i*_out_max_ is the maximum charging and discharging current of the *i*th ESU. Thus, the droop coefficient in Equation (6) is limited by Equation (7).

But with a small droop coefficient, affected by the line impedance, the output voltages of each ESUs are different.

## 4 The second layer controller

### 4.1 The diffusion strategy

Suppose there are *N* nodes in a communication network, each node represents an ESU. By consensus algorithm, the equation for the state variable is expressed as Equation (8).

(8) $$x_{i,k+1}=x_{i,k}+\sum_{j\in N_{i}}a_{ij}\left(x_{j,k}-x_{i,k}\right)=\sum_{j\in N_{i}}a_{ij}x_{j,k}$$

where *i* represents the *i*th node, *k* represent the *k*th iteration. *a*_*ij*_ is the element in adjacent matrix. *N*_*i*_ is the sets of adjacent nodes of the *i*th node.

For the diffusion strategy, the communication topology is the same as the consensus algorithm. While their adjacent matrix is different. In the diffusion strategy, the adjacent matrix is not symmetrical, and *a*_*ij*_ is the weight coefficient that each node places on the others.

If the *j*th node and the *i*th node are adjacent nodes, *a*_*ij*_ is a positive value. If the *j*th node is not the adjacent node of the *i*th node, *a*_*ij*_ is equal to 0.

Literature [27] introduces that *a*_*ij*_ should satisfy Equation (9) to obtain the convergence of distributed optimization.

(9) $$\sum_{i=1}^{N}a_{ij}=1$$

Equation (10) describes a formula for *a*_*ij*_ to obtain a stability and convergence of mutative communication topology by using Metropolis rules. And it is expressed as

(10) $$a_{ij}=\left\{ \begin{array}{l} \frac{1}{\max\left(n_{i},n_{j}\right)}\;i\in N_{j}\backslash \left\{j\right\}\\ 1-\sum_{i\in N_{j}\backslash \left\{j\right\}}a_{ij}\;i=j \end{array}\right.$$

where *n*_*i*_ and *n*_*j*_ are the total number of adjacent nodes of the *i*th node and the *j*th node.

The diffusion strategy divides into two steps, adapt and combine. In the first step, an intermediate variable is calculated by the gradient of the objective function and the information from the adjacent nodes, it is expressed as Equation (11).

(11) $$\varphi_{i,k}=x_{i,k-1}-\mu_{i}\times \nabla J_{i}\left(x_{i,k-1}\right)$$

where *φ*_*i,k*_ is the intermediate variable of the *i*th node at the *i*th iteration. *μ*_*i*_ is step size, and it is a small positive constant. *J*_*i*_(*x*_*i,k*-1_) is the objective function.

In the second step, the state variable is calculated and expressed as Equation (12).

(12) $$x_{i,k}=\sum_{j\in N_{i}}a_{ij}\varphi_{j,k}$$

where, *φ*_*j,k*_ is renewed by Equation (11).

**Fig. 7:**
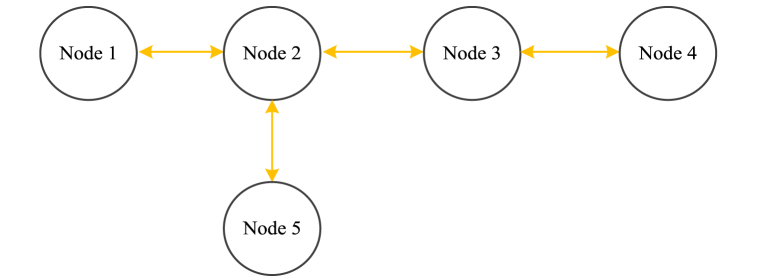
A typical communication topology.

Fig. 7 shows a typical communications topology of five nodes.

For the communication topology shown in Fig. 7, the adjacent matrix of diffusion strategy is expressed as Equation (13), and the adjacent matrix of consensus algorithm is expressed as Equation (14).

(13) $$A_{1}=\left[\begin{array}{ccccc} 3/4 & 1/4 & 0 & 0 & 0\\ 1/4 & 1/4 & 1/4 & 0 & 1/4\\ 0 & 1/4 & 1/4 & 1/2 & 0\\ 0 & 0 & 1/4 & 3/4 & 0\\ 0 & 1/4 & 0 & 0 & 3/4 \end{array}\right]$$

(14) $$A_{2}=\left[\begin{array}{ccccc} 0 & 1 & 0 & 0 & 0\\ 1 & 0 & 1 & 0 & 1\\ 0 & 1 & 0 & 1 & 0\\ 0 & 0 & 1 & 0 & 0\\ 0 & 1 & 0 & 0 & 0 \end{array}\right]$$

The contrast of Equation (8) and Equation (12), and the contrast of Equation (13) and Equation (14) show the essential difference between diffusion strategy and consensus algorithm. Compared with the state variable in consensus algorithm, the diffusion strategy improves the convergence speed by introducing the grant of the objective function. And compared with the adjacent matrix in consensus algorithm, the diffusion strategy introduces a weight factor to reduce the convergence time.

### 4.2 The compensation for line impedance

Since the line impedance causes an output voltage drop, the output voltages of distributed ESUs are different, which results in a circulating current between different ESUs. One of the control objective in second layer controller is the balanced output voltage. So the control objective is equivalent to achieve the minimum mean square error of average output voltage, and it is expressed as $\mathrm{argmin}{{\sigma_{u}}_{i}}^{2}\left(u_{\text{avg}i}\right)$. *u*_avgj_ is the average output voltage of the *j*th node, and expressed as Equation (15). ${{\sigma_{u}}_{i}}^{2}\left(u_{\text{avg}i}\right)$ is the mean square error of *u*_avgi_, and expressed as Equation (16).

(15) $$u_{\text{avg}i}=\sum_{j\in N_{i}}a_{ij}u_{\text{out}j}$$

(16) $${{\sigma_{u}}_{i}}^{2}={\left(u_{\text{avg}i}-\sum_{j\in N_{i}}a_{ij}u_{\text{avg}j}\right)}^{2}={\left(\sum_{j\in N_{i}}a_{ij}u_{\text{out}j}-\sum_{j\in N_{i}}a_{ij}u_{\text{avg}j}\right)}^{2}$$

Thus, by diffusion strategy, in the first step, the intermediate variable is expressed as Equation (17) based on Equation (11).

(17) $${\varphi_{i}}^{1}\left(k+1\right)={x_{i}}^{1}\left(k\right)-u_{i}\nabla {\sigma_{ui}}^{2}\left(k\right)$$

where ${x_{i}}^{1}\left(k\right)$ is the first state variable, and expressed as Equation (18). $\nabla {\sigma_{ui}}^{2}\left(k\right)$ is expressed as Equation (19).

(18) $${x_{i}}^{1}\left(k\right)=u_{\text{avg}i}$$

(19) $$\nabla {\sigma_{ui}}^{2}\left(k\right)={\left(u_{\text{avg}i}\left(k\right)-\sum_{j\in N_{i}}a_{ij}u_{\text{avg}j}\left(k\right)\right)}^{2}-{\left(u_{\text{avg}i}\left(k-1\right)-\sum_{j\in N_{i}}a_{ij}u_{\text{avg}j}\left(k-1\right)\right)}^{2}$$

in the second step, the state variable is updated based on Equation (12), and it is expressed as Equation (20).

(20) $${x_{i}}^{1}\left(k+1\right)=\sum_{j\in N_{i}}{a_{i}}_{j}{\varphi_{i}}^{1}\left(k+1\right)$$

So by the diffusion strategy, the compensation voltage caused by impedance line is expressed as Equation (21).

(21) $$\Delta u_{ui}\left(k\right)=\Delta u_{ui2}\left(k\right)+g_{i}\cdot \Delta u_{ui1}\left(k\right)$$

where $\Delta u_{ui2}\left(k\right)$ is the compensation of the unbalanced output voltage of distributed ESUs, and expressed as Equation (23). $\Delta u_{ui1}\left(k\right)$ is the compensation of the reference voltage, and expressed as Equation (22). If the *i*th node can receive the reference voltage, *g*_*i*_ is equal to 1. If the *i*th node cannot receive the reference voltage, *g*_*i*_ is equal to 0.

(22) $$\Delta u_{ui1}\left(k\right)=k_{Pv1}\left(v_{\text{ref}}-v_{\text{avg}i}\left(k\right)\right)+k_{Iv1}\int \left(v_{\text{ref}}-v_{\text{avg}i}\left(k\right)\right)$$

(23) $$\Delta u_{ui2}\left(k\right)=k_{Pv2}\left({\overset{⌢}{v}}_{\text{avg}i}\left(k+1\right)-v_{\text{avg}i}\left(k\right)\right)+k_{Iv2}\int \left({\overset{⌢}{v}}_{\text{avg}i}\left(k+1\right)-v_{\text{avg}i}\left(k\right)\right)$$

where *k*_Pv1_, *k*_Iv1_ are the proportional coefficient and integral coefficient of reference voltage controller. *k*_Pv2_, *k*_Iv2_ are the proportional coefficient and integral coefficient of average output voltage controller. ${\overset{⌢}{v}}_{\text{avg}i}\left(k+1\right)$ is the estimated value of average output voltage of the *i*th nodes at the *k*th iteration, and expressed as Equation (24).

(24) $${\overset{⌢}{v}}_{\text{avg}i}\left(k+1\right)={x_{i}}^{1}\left(k+1\right)$$

### 4.3 The compensation for equalization SOC

The other objective of the second layer is to allocate the power between distributed ESUs to achieve equalization SOC. The state variable is expressed as Equation (25).

(25) $$x_{i}^{2}=\gamma_{i}=\frac{P_{i}}{C_{i}\cdot S_{i}}$$

where $x_{i}^{2}$ is the second state variable. *P*_*i*_ is the charging and discharging power of the *i*th ESU, and expressed as Equation (26). *C*_*i*_ is the capacity of the *i*th ESU. *S*_*i*_ is the remaining SOC of the *i*th ESU. During the discharging period, *S*_*i*_ is expressed as Equation (27). During the charging period, *S*_*i*_ is expressed as Equation (28).

(26) $$P_{i}=u_{i\_\text{out}}\times i_{i\_\text{out}}$$

(27) $$S_{i}={\text{SOC}}_{i}-{\text{SOC}}_{\min}$$

(28) $$S_{i}={\text{SOC}}_{\max}-{\text{SOC}}_{i}$$

where, SOC_min_ and SOC_max_ are the minimum and maximum SOC of each ESUs, typically 20 % and 80 %.

The control objective is equivalent to obtain the minimum mean square error of the state variable, and it is expressed as $\mathrm{argmin}{{\sigma_{\gamma }}_{i}}^{2}$, and ${{\sigma_{\gamma }}_{i}}^{2}$ is the minimum mean square error of *γ*_*i*_, and is expressed as Equation (29).

(29) $${\sigma_{\gamma i}}^{2}={\left(\gamma_{i}-\gamma_{\text{avg}i}\right)}^{2}={\left(\frac{P_{i}}{C_{i}S_{i}}-\sum_{j\in N_{i}}\frac{P_{j}}{C_{j}S_{j}}\right)}^{2}$$

where, *γ*_avgi_ is the average value of *γ*_j_ of the adjacent nodes, and expressed as Equation (30).

(30) $$\gamma_{\text{avg}i}=\sum_{j\in N_{i}}\gamma_{j}=\sum_{j\in N_{i}}\frac{P_{j}}{C_{j}\cdot S_{j}}$$

Thus, by diffusion strategy, in the first step, the intermediate variable is expressed as Equation (31) based on Equation (11).

(31) $${\varphi_{i}}^{2}\left(k+1\right)={x_{i}}^{2}\left(k\right)-u_{i}\nabla {\sigma_{\gamma i}}^{2}\left(k\right)$$

where $\nabla {\sigma_{\gamma i}}^{2}\left(k\right)$ is expressed as Equation (32).

(32) $$\nabla {\sigma_{\gamma i}}^{2}\left(k\right)={\sigma_{\gamma i}}^{2}\left(k\right)-{\sigma_{\gamma i}}^{2}\left(k-1\right)={\left(\frac{P_{i}\left(k\right)}{C_{i}\left(k\right)\times S_{i}\left(k\right)}-\sum_{j\in N_{i}}\frac{P_{j}\left(k\right)}{C_{j}\left(k\right)\times S_{j}\left(k\right)}\right)}^{2}-{\left(\frac{P_{i}\left(k-1\right)}{C_{i}\left(k-1\right)\times S_{i}\left(k-1\right)}-\sum_{j\in N_{i}}\frac{P_{j}\left(k-1\right)}{C_{j}\left(k-1\right)\times S_{j}\left(k-1\right)}\right)}^{2}$$

in the second step, the state variable is updated and expressed as Equation (33) based on Equation (12).

(33) $${x_{i}}^{2}\left(k+1\right)=\sum_{j\in N_{i}}{a_{i}}_{j}{\varphi_{i}}^{2}\left(k+1\right)$$

Thus, the compensation voltage for equalization SOC by diffusion strategy is expressed as Equation (34).

(34) $$\Delta u_{\gamma i}\left(k\right)=k_{P\gamma }\left({\overset{⌢}{\gamma }}_{i}\left(k+1\right)-\gamma_{i}\left(k\right)\right)+k_{I\gamma }\int \left({\overset{⌢}{\gamma }}_{i}\left(k+1\right)-\gamma_{i}\left(k\right)\right)$$

where $\Delta u_{\gamma i}\left(k\right)$ is the compensation voltage to allocate power to distributed ESUs balanced. ${\overset{⌢}{\gamma }}_{i}\left(k+1\right)$ is the estimated value of *γ*_*i*_ at the *k*th iteration, and expressed as Equation (35). *k*_P*γ*_ and *k*_I*γ*_ are the proportional coefficient and integral coefficient of *γ*_*i*_ controller.

(35) $${\overset{⌢}{\gamma }}_{i}\left(k+1\right)={x_{i}}^{2}\left(k+1\right)$$

### 4.4 The second layer controller

The control framework of distributed ESUs is divided into two layer, the first layer adopts the improved droop control with adaptive droop coefficients, and the second layer controller applies diffusion strategy. The second layer controller contains two parts, the one part is to compensate the voltage deviation caused by the droop control and line impedance, the other part is to allocate power balanced for equalization SOC.

**Fig. 8:**
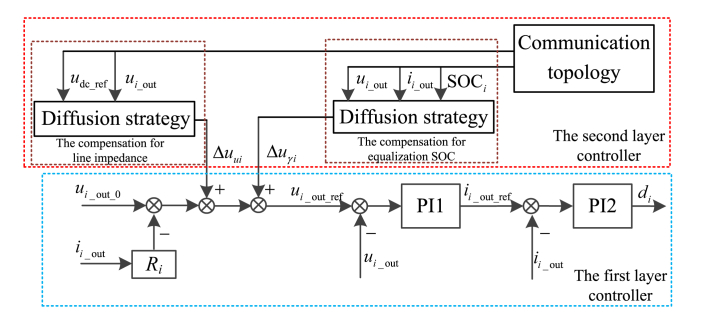
The two layer controller.

Fig. 8 shows the two layer controller for distributed ESU.

As shown in Fig. 8, the two layer controller requires the communication network system, while once the communication network system is down, with the first layer controller, ESU can still operates with improved droop control.

## 5 Results

### 5.1 A the improved droop control

To verify the effectiveness of the improved droop control in the first layer controller, a simulation model with two ESUs is built. The voltage of battery is 200 V, the DC bus voltage is 400 V. The capacity of each ESUs are 1 Ah. The initial SOC of the first ESU is 40 %, the initial SOC of the second ESU is 50 %. At the first stage, the load power is 10 kW, at the second stage, the load power is 25 kW, and the third stage, the load power is 15 kW, the output power of PV is 20 kW. The constant droop coefficient is 5, the equalizing coefficient of SOC is 2. The proportional coefficient and integral coefficient of inner loop are 0.4 and 10, the proportional coefficient and integral coefficient of outer loop are 0.9 and 15, the line impedance is 0.02Ω.

**Fig. 9:**
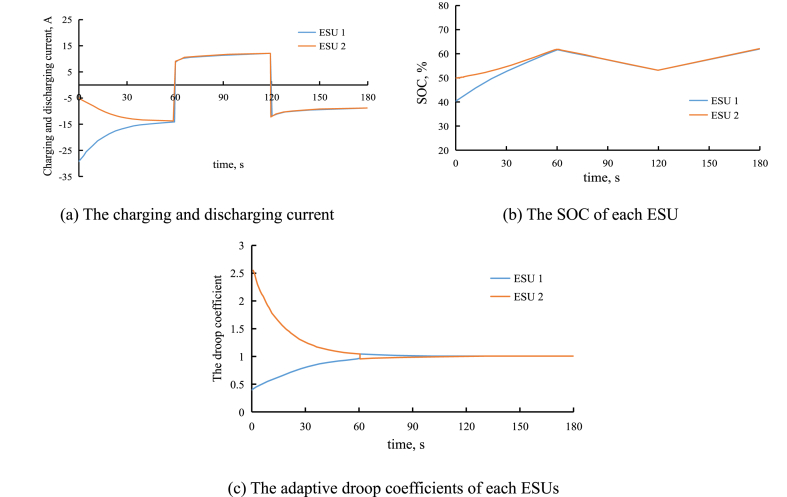
The simulation result of improved control strategy.

With the improved droop control, Fig. 9(a) shows the charging and discharging current of each ESUs, Fig. 9(b) shows the SOCs of each ESUs, and Fig. 9(c) shows the droop coefficients of each ESUs.

As shown in Fig. 9 (c), since the initial SOC of the second ESU is smaller, its droop coefficient is smaller during the charging period, and droop coefficient is larger during the discharging period. Thus, as shown in Fig. 9 (a), for the second ESU, the discharging power is smaller, and the charging power is larger. Conversely, for the first ESU, the discharging power is larger, and the charging power is smaller. And as shown in Fig. 9 (b), the balanced SOC of each ESU is achieved within minutes.

### 5.2 The diffusion strategy

**Fig. 10:**
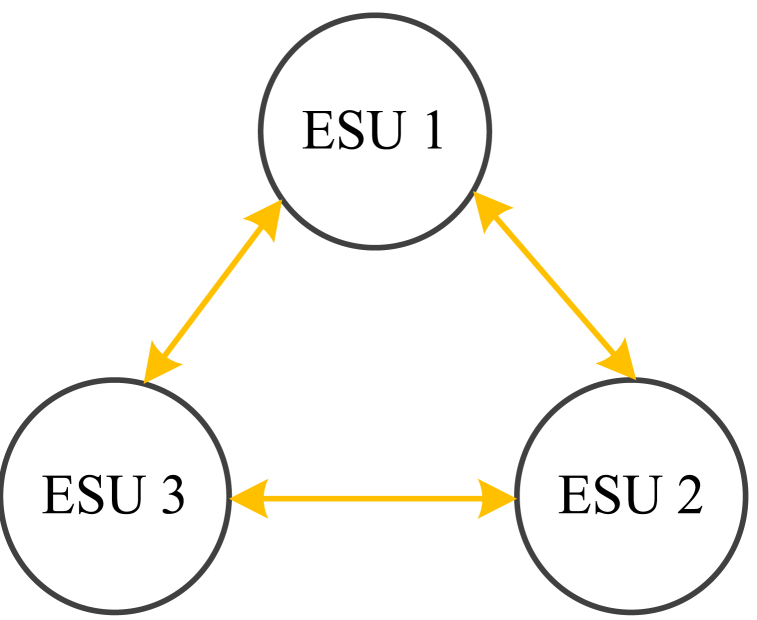
The communication of distributed ESUs.

To verify the effectiveness of diffusion strategy for distributed ESUs in the second layer controller, a simulation model with 3 ESUs is built, and the initial SOC of each ESU is 55 %, 45 % and 33 %. And the communication topology is shown in Fig. 10.

**Fig. 11:**
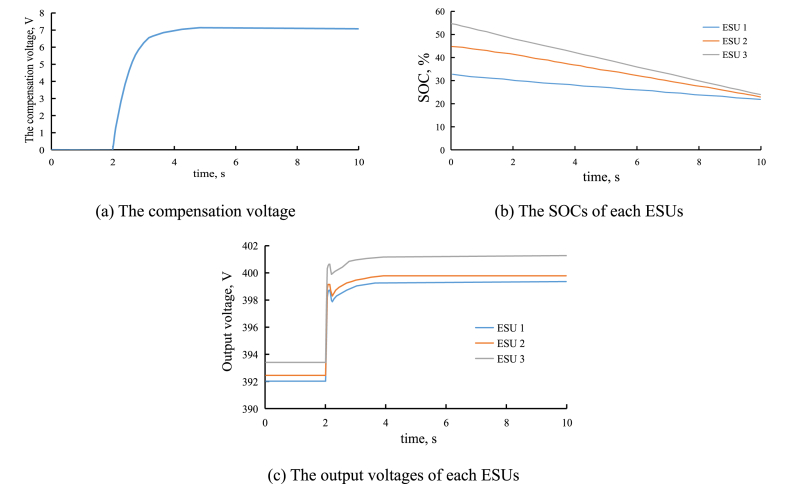
The results by diffusion strategy.

At 0s–2s, only the improved droop control is used to the control ESU, and at 2s, the second layer controller is introduced. Fig. 11 (a) shows the voltage compensation of output voltage analyzed by diffusion strategy, Fig. 11 (b) shows the SOCs of each ESUs, and Fig. 11 (c) shows the output voltages of each ESUs.

As shown in Fig. 11(a), without the second layer controller, the compensation voltage is equal to 0 V, and with the second layer controller, the compensation voltage is updated with the number of iterations.

Compared Fig. 11, Fig. 10, with the second layer controller, the rate of SOC equalization is higher. Without the second layer controller, it takes about 60s to realize SOC equalization. While with the second layer controller, it takes only about 10s to realize SOC equalization. In other words, the convergence rate of SOC increases, so the convergence time decreases.

As shown in Fig. 11(c), with the second layer, the output voltage of ESU is around 399 V. And without the second layer, the output voltage of ESU is around 394 V. The reference output voltage is set to 400 V. Thus, with the second layer controller, the steady-errors of output voltages of each ESUs are smaller.

### 5.3 The diffusion strategy and decentralized control

**Fig. 12:**
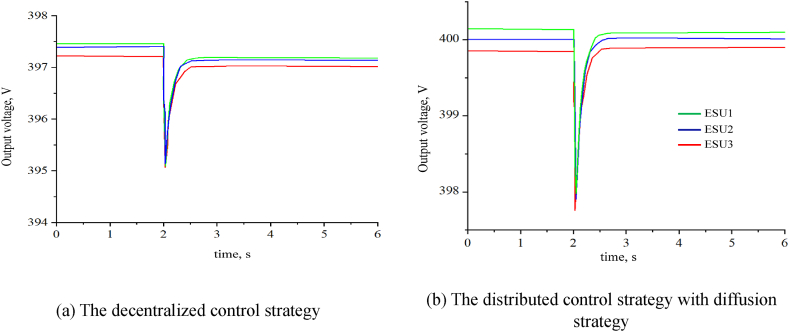
The output voltage of ESUs with different control strategy.

In this simulation model, the parameters of ESUs are the same as the parameters in the second simulation model. At 0s, the impedance is set to 20Ω, and at 2s, the impedance is set to 15Ω. Fig. 12(a) shows the output voltage of ESUs with the decentralized control strategy, and Fig. 12(b) shows the output voltage of ESUs with diffusion strategy.

As shown in Fig. 12(a), with the decentralized control strategy, ESUs can output a stable voltage with a steady state error, and the steady state error increases with the output current. As shown in Fig. 12(b), withe the distributed control strategy, ESUs can also output a stable voltage without steady state error. Thus, with the diffusion strategy, the circulating current between different ESUs decreases.

## 6 Conclusions

A two layer controller is proposed for the ESS using the distributed diffusion strategy. The first layer controller is an improved droop control with adaptive droop coefficients. And the second layer applies diffusion strategy to compensate the voltage deviation. The voltage deviation compensation contains two parts, one is to compensate the voltage deviation caused by droop control and line impedance. The other one is to allocate the charging and discharging power to achieve a balanced SOC.

In the second controller with diffusion strategy, the control objective is equivalent to an objective function, and the distributed diffusion strategy is used to solve the optimization problem. Compared with consensus algorithm, by introducing the gradient of objective function, the convergence rate increases. And the results shows that the proposed strategy offers a superior performance on the distributed ESUs.

## CRediT authorship contribution statement

**Zejian Liu:** Validation, Methodology, Investigation, Conceptualization. **Ping Yang:** Resources, Funding acquisition, Conceptualization. **Xu Lin:** Supervision, Software, Project administration. **Ziwei Fan:** Visualization, Data curation, Conceptualization. **Cencen Hong:** Writing – review & editing, Writing – original draft, Methodology, Investigation.

## Data availability statement

The material about simulation model related to this article can be found in the supplementary materials. Also, they can be found at https://pan.quark.cn/s/0706b08a06c2.

## Funding

Ping Yang reports financial support was provided by The Marine Economic Development (Six Marine Industries) Special Fund Project of Guangdong Province (No. GDNRC[2023]27).

## Declaration of competing interest

The authors declare the following financial interests/personal relationships which may be considered as potential competing interests:Ping YANG reports financial support was provided by The Marine Economic Development (Six Marine Industries) Special Fund Project of Guangdong Province. If there are other authors, they declare that they have no known competing financial interests or personal relationships that could have appeared to influence the work reported in this paper.
